# Bioadhesive Chitosan Films Loading Curcumin for Safe and Effective Skin Cancer Topical Treatment

**DOI:** 10.3390/pharmaceutics17010018

**Published:** 2024-12-26

**Authors:** Seila Tolentino, Mylene M. Monteiro, Felipe Saldanha-Araújo, Marcilio Cunha-Filho, Tais Gratieri, Eliete N. Silva Guerra, Guilherme M. Gelfuso

**Affiliations:** 1 Laboratory of Food, Drugs, and Cosmetics (LTMAC), University of Brasilia, Brasília 70910-900, Brazil; seilatolentino@hotmail.com (S.T.); marciliofarm@hotmail.com (M.C.-F.); tgratieri@gmail.com (T.G.); 2 Laboratory of Oral Histopathology, University of Brasilia, Brasília 70910-900, Brazil; mylenemonteiro7@gmail.com (M.M.M.); elieteneves@unb.br (E.N.S.G.); 3 Laboratory of Hematology and Stem Cells (LHCT), School of Health Sciences, University of Brasília, Brasília 70910-900, Brazil; felipearaujo@unb.br

**Keywords:** cancer, drug delivery, bioadhesive film, skin, topical delivery

## Abstract

**Background/Objectives**: This study aimed to evaluate the safety and efficacy of chitosan-based bioadhesive films for facilitating the topical delivery of curcumin in skin cancer treatment, addressing the pharmacokinetic limitations associated with oral administration. **Methods**: The films, which incorporated curcumin, were formulated using varying proportions of chitosan, polyvinyl alcohol, Poloxamer^®^ 407, and propylene glycol. These films were assessed for stability, drug release, in vitro skin permeation, cell viability (with and without radiotherapy), and skin irritation. **Results**: The films demonstrated physical stability and preserved curcumin content at room temperature for 90 days. Drug release was effectively controlled during the first 8 h, with release rates ranging from 51.6 ± 4.8% to 65.6 ± 13.0%. The films also enhanced drug penetration into the skin compared to a curcumin solution used as a control (stratum corneum: 1.3 ± 0.1 to 1.9 ± 0.8 µg/cm²; deeper skin layers: 1.7 ± 0.1 to 2.7 ± 0.2 µg/cm²). A cytotoxicity test on metastatic melanoma cells showed that curcumin at topical doses exerted activity similar to that delivered via the skin. Furthermore, curcumin alone was more effective in inhibiting tumor cells than radiotherapy alone (*p* < 0.01), with no additional benefit observed when curcumin was combined with radiotherapy. Finally, irritation tests confirmed that the films were safe for topical application. **Conclusion**: The developed chitosan-based bioadhesive films represent a promising alternative for the topical treatment of skin tumors using curcumin.

## 1. Introduction

According to the International Agency for Research on Cancer, non-melanoma skin cancer accounts for over a million new cases worldwide (excluding basal cell carcinoma) [1]. On the other hand, melanoma-type skin tumors, despite being less prevalent, corresponding to only 3% of malignant skin neoplasms, are the most severe and lethal cutaneous cancer type due to their high likelihood of metastasis [2,3].

Skin cancer is associated with a combination of genetic and environmental factors, with 90–95% attributed to UV exposure [3,4,5]. Other risk factors include exposure to arsenic, immunosuppression, and viral infections (such as human papillomavirus) [2,3,5,6].

Notwithstanding surgical excision being the standard for treating epithelial tumors, adjuvant therapies like chemotherapy are also demanded. However, these adjuvant treatments involve high levels of toxicity, drug resistance, and recurrences [7,8]. Thus, the importance of developing new therapeutic strategies that are safer, effective, and affordable, such as using phytochemicals, is evident. Polyphenols, such as curcumin, are natural compounds that have shown effects against tumoral cells [8,9].

In fact, numerous studies have published evidence of curcumin’s action against various tumors, including melanoma, breast, head and neck, prostate, and ovarian cancers [10]. This drug affects the expression of the nuclear factor NFκB, which regulates cell proliferation, metastasis, angiogenesis, apoptosis, and resistance to chemotherapy. Additionally, curcumin inhibits key signaling pathways involved in cell proliferation, including PI3K, AKT, mTOR, AP1 (JUN and FOS), JNK, JAK-STAT, PKC, CMYC, MAPK, ELK, CDKs, iNOS, and Wnt/β-catenin. In this way, by targeting multiple cell proliferation pathways, curcumin helps prevent cancer promotion [11].

Curcumin is one of the primary compounds found in the rhizome of Curcuma longa [12]. For centuries, turmeric has been used in traditional Chinese medicine as a spice, food coloring, and medicinal agent. Curcumin belongs to the curcuminoid family, which includes two other compounds: demethoxycurcumin and bisdemethoxycurcumin. The chemical structure of curcumin, [(1E,6E)-1,7-bis(4-hydroxy-3-methoxyphenyl)hepta-1,6-diene-3,5-dione], exhibits keto-enol tautomerism [13]. This drug is also chemically unstable, rapidly degrading at physiological pH due to alkaline hydrolysis or auto-oxidation [14]. However, it has physical–chemical limitations resulting in low oral bioavailability, poor absorption, and rapid systemic elimination [15]. These challenges highlight the need for safer pharmaceutical forms and administration routes for targeted applications.

Bioadhesive films are designed to carry the drug and deliver it directly to the target site. They maintain the formulation on the biological surface where its action occurs, thereby increasing local bioavailability [16,17]. Such a drug delivery avoids pre-systemic metabolism in the gastrointestinal tract, reduces the drug’s first-pass effect, controls drug release for an extended period, facilitates application and removal in unconscious or traumatized patients, allows for the application of permeation enhancers, and offers flexibility in shape and size [18].

Bioadhesion results from various mechanisms, typically divided into two stages: the first is a contact phase, where the film comes into proximity with the tissue, and the second is a consolidation stage, where physical–chemical interactions occur to strengthen prolonged adhesion [18,19,20,21]. Film adhesion can occur through ionic bonds, covalent bonds, hydrogen bonds, or van der Waals forces [19,20,21]. The intensity and characteristics of these interactions can be modified according to the polymer used.

Chitosan is an extensively studied natural polymer in preparing bioadhesive films [22]. It is a cationic polysaccharide formed by the partial N-deacetylation of chitin, a substance found in the exoskeleton of crustaceans, fungi, and insects [23]. Chitosan is biocompatible and biodegradable, with promising characteristics for sustained drug release, mucoadhesive properties, and increased skin penetration [16,17,22,23]. This last characteristic is essential for skin application since curcumin is a very lipophilic molecule, log P = 3.29 [24], which would have difficulty crossing the first layer of the epidermis—the stratum corneum, an equally very lipophilic barrier.

Thus, this study proposes using chitosan-based bioadhesive films loaded with curcumin to address the pharmacokinetic challenges of this drug in the quest for an effective and safe topical alternative for skin cancer treatment. The development and physical–chemical characterization of the films have already been presented in previous work [25]. In this study, we verified the physicochemical stability of the film and compared in vitro the release rate and skin permeation of the drug from them. Curcumin activity in a resistant melanoma cell model was also performed to determine the toxic dose of the drug. Finally, we evaluated the product’s safety for topical application using a non-animal methodology.

## 2. Material and Methods

### 2.1. Chemicals and Reagents

Curcumin (>96.0%) was obtained from Florien (Rio de Janeiro, Brazil). Medium-molecular-weight chitosan, polyvinyl alcohol (PVA), Poloxamer^®^ 407, mucin, acetic acid, RPMI-1640, and MTT (3-(4,5-dimethylthiazol-2yl)-2,5-diphenyl tetrazolium bromide) were purchased from Sigma-Aldrich (Steinheim, Germany). Propylene glycol, Tween^®^ 80, isopropanol, sodium hydroxide, dimethyl sulfoxide, and EDTA were purchased from Dinâmica Química Contemporânea Ltda. (São Paulo, Brazil). Monobasic sodium phosphate, dibasic sodium phosphate, HEPES buffer, and hydrochloric acid were sourced from Vetec (Rio de Janeiro, Brazil). Scotch no. 845 Book Tape was purchased from 3M (St. Paul, MN, USA). Fetal bovine serum (FBS), penicillin, streptomycin, and trypsin were acquired from Gibco (Grand Island, NE, USA). Pre-cleaned filters with a diameter of 22 mm and a pore size of 0.45 μm, both hydrophobic and hydrophilic, were obtained from Analítica (São Paulo, Brazil). All experiments used ultrapure water (Millipore, Illkirch-Graffenstaden, France).

Skin from porcine ears was obtained from a local abattoir—Via Carnes (Formosa, Brazil) and used full-thickness. The ear’s outer portion had its entire skin removed, carefully separated from the underlying layer. Subsequently, the skin was stored in a frozen state at −20 °C for a maximum period of 1 month before use. In this case, there is no need for approval since, in Brazil, porcine skin is approved for food consumption.

### 2.2. Preparation of the Films

As previously described, films were obtained using the technique of solvent evaporation from aqueous polymeric dispersions [25]. Film F1 was prepared with the following composition: chitosan, propylene glycol, PVA (0.5:5.0:8.5%, respectively), film F2: chitosan, Poloxamer^®^ 407, PVA (0.5:1.0:8.5%, respectively), and film F3: chitosan, Poloxamer^®^ 407, propylene glycol, PVA (0.5:1.0:5.0:8.5%, respectively). They were prepared from a 1 mg/mL curcumin solution in ethanol, reaching 78.6 µg/cm^2^ in the film [25].

### 2.3. Stability of the Films

The stability of the films was assessed by storing them in sealed aluminum sachets at both room temperature and 40 ± 3 °C (*n* = 3 for each film and storage condition). The appearance, weight, thickness, pH, and curcumin content were analyzed at predetermined intervals of 0, 7, 30, 60, and 90 days. After each period, the samples were extracted from the packaging, photographed, and subjected to analysis based on the procedure specific to each defined parameter as previously described [25].

A temperature of 40 °C was used because it is recommended for accelerated stability testing according to the Food and Drug Administration’s Q1A(R2) guidelines on the Stability Testing of New Drug Substances and Products.

### 2.4. Drug Release

The film’s curcumin release profile was obtained using a Phoenix DB-6 diffusion system (Teledyne Hanson, Chatsworth, CA, USA) coupled to vertical diffusion cells (diffusional area = 1.5 cm^2^). The films (F1, F2, and F3) were placed between the donor and the receptor compartments of the cells (*n* = 5 for each formulation). The receptor compartment was filled with 15 mL pH 5.5 phosphate buffer with 0.5% Tween ^®^ 80 (medium chosen to ensure sink conditions for the release test while also preventing drug degradation, as curcumin is sensitive to physiological pH), which was kept under magnetic stirring (300 rpm) at 37 °C. The test was monitored for 24 h with the collection of receiver solution samples at 1, 2, 4, 6, 8, 18, 20, 22, and 24 h. The concentration of released curcumin was analyzed by HPLC (Section 2.8), and the release profiles were obtained by plotting the percentage of the released drug (%) as a function of time (h).

### 2.5. In Vitro Skin Permeation

Films F1, F2, and F3 soaked in 100 µL of purified water were positioned on the surface of the skin obtained from porcine ears, in contact with the stratum corneum (diffusional area of 1.77 cm^2^). The skin separated the donor and receptor compartments. This last compartment contained 15 mL pH 5.5 phosphate buffer with 0.5% Tween^®^ 80 (to ensure sink conditions). The diffusion cells were maintained at 37 ± 3 °C under magnetic stirring (300 rpm) for 24 h (*n* = 5 for each formulation). As a control, the donor compartment was filled with 100 µL of a curcumin solution at 139 µg/mL in pH 5.5 phosphate buffer with 0.5% Tween^®^ 80.

At the end of each series of experiments, 1 mL of the receptor solution was gathered and assessed for curcumin content. Then, the skin was placed on a flat surface, with the stratum corneum facing up. After, the stratum corneum was removed with the successive application of 15 adhesive tapes. These strips were placed in an amber glass container, and then the remaining skin was cut into small fragments and placed in other amber glass containers. The curcumin extraction process from the skin and quantification using HPLC followed a previously validated methodology [26] described below (Section 2.8).

### 2.6. Cell Viability Assays

#### 2.6.1. Cell Culture

Cell viability experiments used the metastatic melanoma cell line MeWo (ATCC). Cells were cultured in RPMI 1640 medium supplemented with 10% FBS and 1% penicillin/streptomycin at 37 °C and 5% CO_2_.

The cells were seeded in 96-well plates at 1 × 10^4^ cells/well density and incubated with 5% CO_2_ at 37 °C for 24 h. Then, they were treated for 24 h with vehicle (DMSO) at the same concentration related to the highest concentration of curcumin and increasing concentrations of curcumin (10.0, 20.0, 30.0, 50.0, 100.0, and 150.0 µg/mL), obtained from a curcumin stock solution at 5 mg/mL in DMSO. After, 10 µL of MTT at 5 mg/mL was added to each well, and the plates were incubated at 37 °C and 5% CO_2_ for 4 h. After this period, the culture media containing the treatment and the MTT solution were carefully aspirated. The crystals formed from the conversion of MTT to formazan were diluted in 100 µL of isopropanol acidified with hydrochloric acid and the plate was homogenized for 15 min. The absorbance was measured at 570 nm using a microplate reader (Multimode Detector, DTX 800, Beckman Coulter, Brea, CA, USA). The dose–response curve produced the computation of half-maximal inhibitory concentrations (IC_50_).

#### 2.6.2. Cell Viability with Radiotherapy

The cells were cultured following the procedure outlined in Section 2.6.1 and treated with curcumin concentrations corresponding to the IC_50_ to evaluate curcumin cytotoxicity in combination with irradiation. After 1 h, the cells treated or not with curcumin were exposed to 4, 8, and 12 Gy of ionizing radiation, utilizing a 6 MV photon linear beam from a Siemens Primus linear accelerator (Malvern, PA, USA), aided by solid water plates. Following 24 h of irradiation, the MTT test was conducted as detailed in Section 2.6.1.

### 2.7. Irritation Assays

Initially, the irritancy levels of films (F1, F2, and F3) were evaluated through the Hen’s Egg Test-Chorioallantoic Membrane (HET-CAM), employing fertilized hen’s eggs acquired from a poultry farm and utilized on the 10th day of fertilization [27]. Initially, the eggshell around the air chamber was removed, and the outer membrane was carefully removed, leaving the chorioallantoic membrane exposed. Then, 300 µL of the samples (films F1, F2, and F3 at 1% in water), positive (1 mol/L NaOH solution), and negative (PBS) controls were applied to this membrane. After 20 s of exposure, the region was washed with saline solution and observed for 5 min (at times 20 s, 2 min, and 5 min) regarding the appearance of any of the following phenomena: hyperemia, hemorrhage, and coagulation, for which a score was given (*n* = 3). In hyperemia, dilated vessels can be observed; in hemorrhage, blood extravasation is evident; and in coagulation, blood clots are visible.

The toxicity of chitosan in HaCaT cells was also evaluated. Cell cultivation and MTT tests were carried out according to item 2.6.1, and the following concentrations of chitosan were used: 0.3, 0.5, 1.0, 2.0, 5.0, and 12.0 mg/mL. Cytotoxicity was classified according to the values found for cell viability, being considered severe for viability below 30%, moderate for values between 30 and 60%, mild for values between 60 and 90%, or non-cytotoxic for values above 90% [28].

### 2.8. HPLC Analysis

Drug quantification was performed on HPLC (LC-20AT, Shimadzu, Kyoto, Japan), using a C_18_ reversed-phase column measuring 250 mm × 4.6 mm × 5 µm (Discorery^®^, Supelco, Germany) as the stationary phase, and phosphoric acid pH 3.0 at 1.0 mmol/L and acetonitrile (47:53) as the mobile phase at a flow rate of 1.0 mL/min. The column oven was maintained at 40 °C. Curcumin was detected at 424 nm, and the injection volume of each sample was 10 μL. Data acquisition, analysis, and reporting were performed using Shimadzu LC software. This method was previously validated regarding selectivity, linearity, detection and quantitation limits (LOD and LOQ, respectively), precision, accuracy, and robustness [26].

### 2.9. Data Analyses

The GraphPad Prism 6 program was employed for statistical analysis of the data. As data resulted in parametric distribution by the Shapiro–Wilk test, a comparative analysis was performed by ANOVA, followed by Tukey’s post-test. The Student’s *t*-test was used to compare samples regarding stability, drug release, and permeation tests. The Kruskal–Wallis test was used for cell viability data, and IC_50_ was calculated after nonlinear regression on dose–response curves. The results were shown in bar graphs represented with mean and standard deviation. The level of statistical significance was accepted as *p* < 0.05.

## 3. Results

### 3.1. Preparation of the Films

The obtained films F1, F2, and F3 were smooth, flexible, and precipitate-free, with uniform weight and thickness, pH-compatible with the skin, resistant to traction, and entrapped curcumin in a high proportion. As previously described, they also exhibited the necessary swelling and mucoadhesion for tissue adherence [25].

### 3.2. Stability of the Films

**Figure 1:**
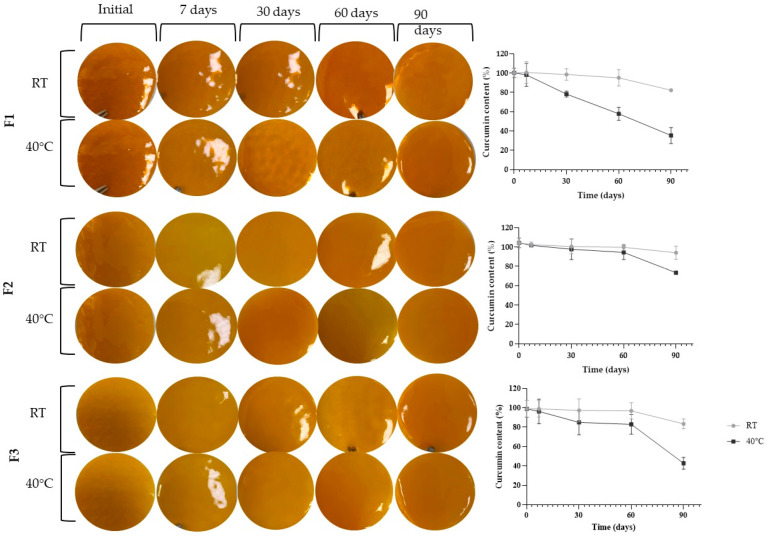
Images captured from the F1, F2, and F3 films at the predefined times for the stability study (0, 7, 30, 60, and 90 days) and variation in curcumin content over time. The storage conditions were room temperature (RT) and 40 °C.

Figure 1 demonstrates the stability results regarding the films’ appearance and curcumin content, while Table 1 shows the films’ weight, thickness, and pH.

As shown in Figure 1, it was observed that over the 90 days, there were no changes in the appearance of the films. Indeed, all remained free of cracks, bubbles, and precipitates without any signs of color or consistency alteration, regardless of the evaluated formulation or temperature. Moreover, no significant changes (*p* > 0.05) were observed regarding weight, thickness, and pH throughout the study. These results indicate the physical stability of the formulation for at least 90 days.

Regarding the curcumin content at room temperature, only film F1 showed significant drug degradation of 16.7% compared to the initial content (*p* > 0.05) at 90 days. The drug content in films F2 and F3 remained stable during this same period.

On the other hand, formulations stored at 40 °C showed higher drug degradation than those stored at room temperature. Film F1 exhibited significant degradation from 30 days, with a degradation of 22.0%. At 60 days, the degradation was 42.3%, and at 90 days, it reached 64.8%. For film F2, curcumin degradation was only significant at 90 days, reaching 29.5%. The same occurred for film F3, with a degradation of 56.8% at 90 days of the experiment.

### 3.3. Drug Release

**Figure 2:**
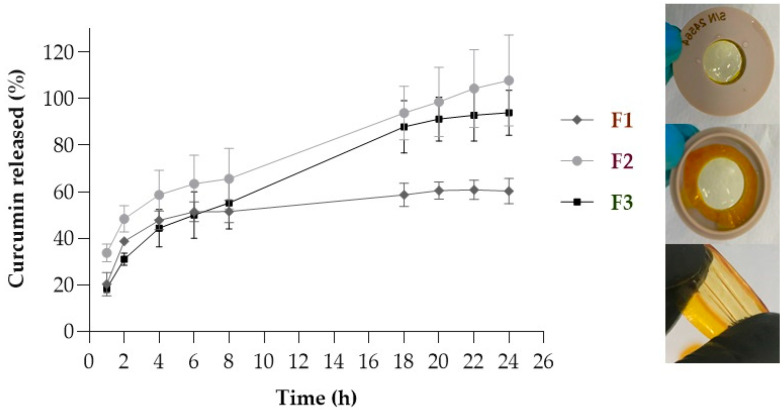
Curcumin release profiles from films (F1, F2, and F3) over 24 h, with collections at 1, 2, 4, 6, 8, 18, 20, 22, and 24 h and recording photographs of the films at the end of the study.

Figure 2 shows the curcumin release profiles from each film and the films’ integrity at the end of the experiment.

Film F1 released curcumin in a sustained manner, reaching approximately 60% drug release at 24 h. Films F2 and F3, in turn, released more than 94% of the drug in the same period. One of the probable reasons for this behavior is related to the degree of swelling of the films since the F2 and F3 films showed more significant swelling than the F1 film [25]. The increased swelling observed in films F2 and F3 compared to film F1 may be related to the presence of Poloxamer. This copolymer has amphiphilic characteristics, which means it tends to form structures from its hydrophobic and hydrophilic chains, thereby facilitating the hydration of the film [17,29]. Swelling is an essential parameter for drug release, as excess swelling can loosen the polymeric network and accelerate drug release [30].

### 3.4. In Vitro Skin Permeation

**Figure 3:**
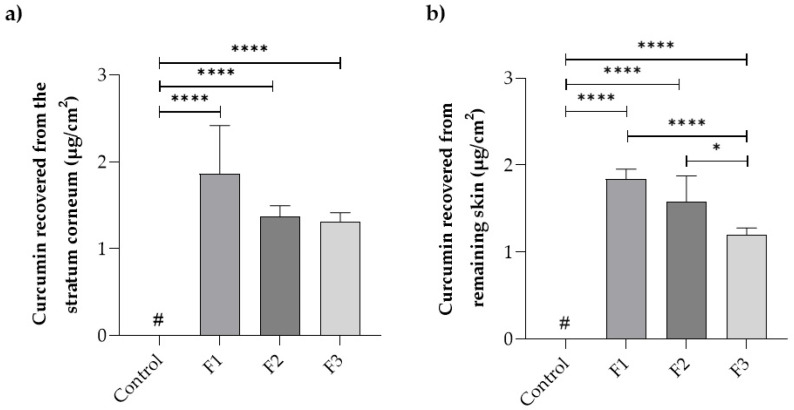
Curcumin recovered from the skin layers (µg/cm^2^) after a 24 h treatment with the films compared to the control. (**a**) Stratum corneum, (**b**) remaining skin. The data represent the mean of 5 determinations ± standard deviation. #, values below the limit of quantification; (*), *p* ≤ 0.05; and (****), *p* ≤ 0.0001.

Figure 3 presents data on the drug distribution in the skin layers (stratum corneum and remaining skin) after a 24 h treatment with the films and the control drug solution.

Curcumin deposition in the first epidermal layer (stratum corneum) was 1.9 ± 0.8 µg/cm^2^ for film F1, 1.4 ± 0.1 µg/cm^2^ for film F2, and 1.3 ± 0.1 µg/cm^2^ for film F3, which were not statistically different (*p* > 0.05) from each other. On the other hand, the control drug solution did not promote curcumin penetration in concentrations above the quantification limit of the method, i.e., 0.4 µg/mL. A similar situation occurred in the remaining skin, where the control solution did not reach the quantification limit. Curcumin penetration in these layers was 2.7 ± 0.2 µg/cm^2^ for film F1, 2.3 ± 0.4 µg/cm^2^ for film F2, and 1.7 ± 0.1 µg/cm^2^ for film F3, with statistical differences (*p* > 0.05) between films F1 and F2 compared to F3.

### 3.5. Cell Viability

**Figure 4:**
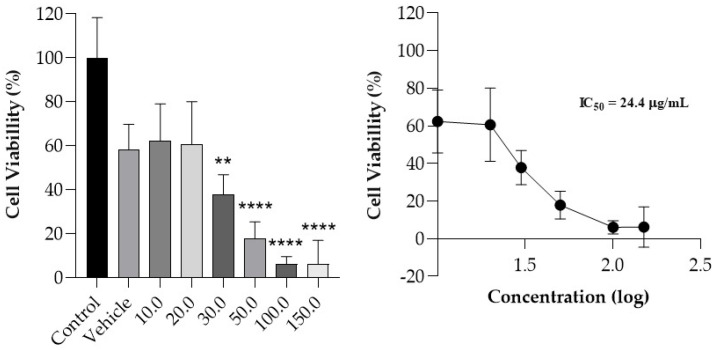
Effect of curcumin on the viability of MeWo cell line. The Kruskal–Wallis test was employed for cell viability data, and IC_50_ values were calculated following nonlinear regression on dose–response curves. The data are presented as the mean ± standard deviation (*n* = 9). (**), *p* ≤ 0.01; and (****), *p* ≤ 0.0001.

The viability of MeWo cells treated with increasing concentrations of curcumin (10.0 to 150.0 µg/mL) was determined, and a dose–response curve was obtained (Figure 4). Compared to the control, a statistically significant decrease in cellular viability (*p* < 0.05) was observed from the 30.0 µg/mL concentration. Curcumin exhibited a dose-dependent effect, with an IC_50_ value of 24.4 µg/mL. It is noted that the IC_50_ value obtained is consistent with the average curcumin concentration that is delivered in deeper skin layers, considering a film with an area of 10 cm^2^ applied to the skin for 24 h.

**Figure 5:**
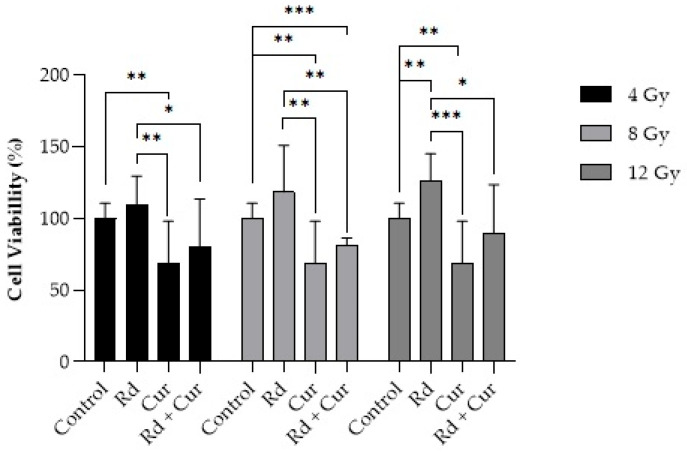
Effect of curcumin on the viability of MeWo cell line, with or without three different doses of radiotherapy. The data are presented as the mean ± standard deviation (*n* = 9). Statistical analysis was performed using ANOVA. (*), *p* ≤ 0.05; (**), *p* ≤ 0.01; and (***); *p* ≤ 0.001.

Regarding the treatment with curcumin combined with radiation doses (4, 8, or 12 Gy, Figure 5), cellular viability decreased compared to radiation alone at the exact dosages, except at doses of 4 Gy, where there was no statistical difference.

### 3.6. Irritability Test

**Figure 6:**
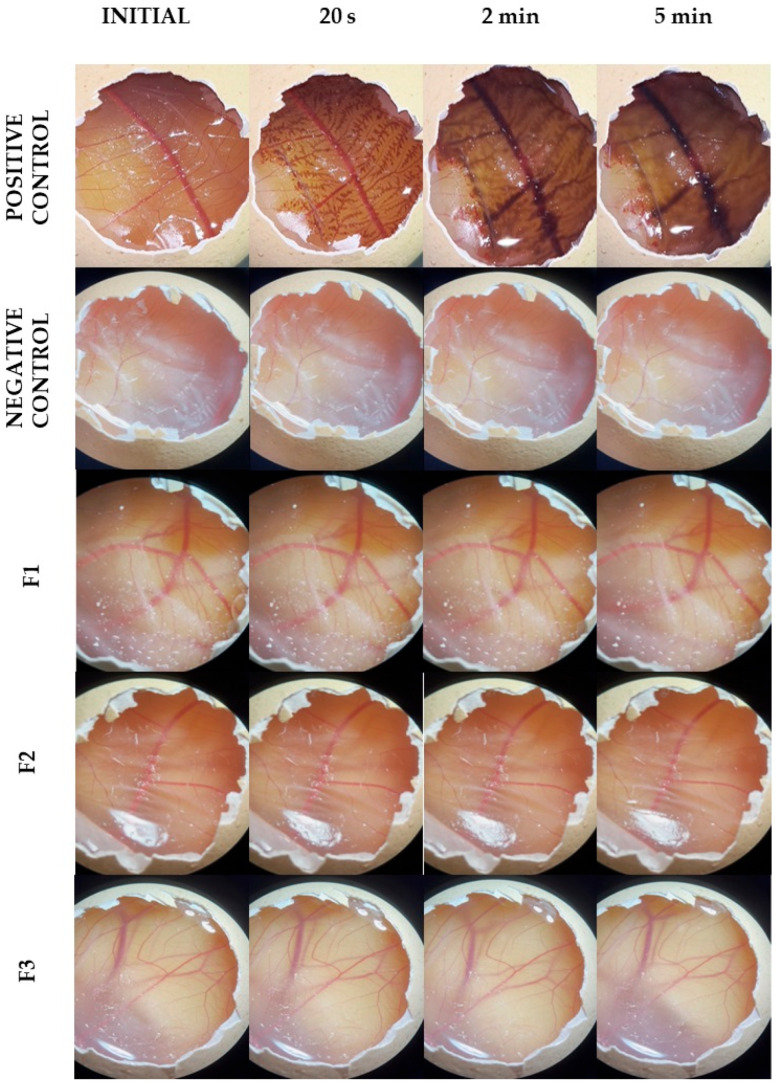
Illustrative sequence of photographic records taken during the HET-CAM assay demonstrating the effects with positive control (1.0 mol/L NaOH), negative control (PBS), and solutions containing each of the films (F1, F2, and F3) solubilized in water on the chorioallantoic membrane after 30 s, 2 min, and 5 min of application.

The images of the CAM presented in Figure 6 were captured just before applying the formulation and 30 s, 2 min, and 5 min after the exposure to the formulations. Using the positive control, changes such as the visualization of capillaries that were not previously visible, dilation of already visible vessels (hyperemia), diffuse blood leakage from vessels and capillaries (hemorrhage), disruption of blood flow in vessels (coagulation), and the appearance of total or partial membrane opacity (opacity) were observed [31].

Notably, the negative control (PBS) did not exhibit these vascular events during the test period. Samples F1, F2, and F3 had a very similar effect to the negative control, with no occurrence of these vascular events during the assay period.

The irritability index of the positive control was 19.0 ± 0.2, while the indices of the negative control and formulations (F1, F2, and F3) were 0.1 ± 0.0. Thus, the positive control was classified as severely irritating, while the negative control and formulations were classified as non-irritating.

**Figure 7:**
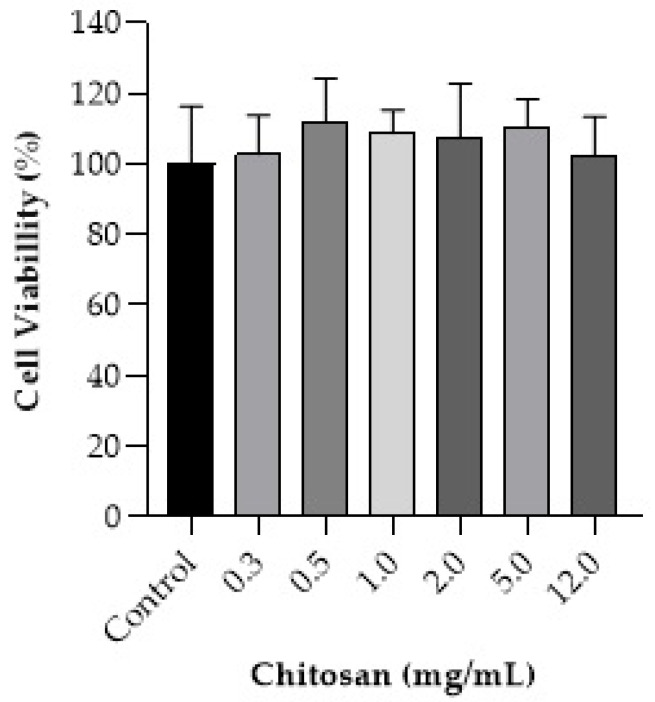
Effect of chitosan on the viability of the human keratinocytes (HaCaT cell line). The Kruskal–Wallis test was employed for cell viability data. Due to the sustained cell viability, it was not possible to calculate the IC_50_ from the obtained dose–response curve. The data are presented as the mean ± standard deviation (*n* = 9).

Figure 7 presents the toxicity profile of chitosan in human keratinocytes, representing the primary mucoadhesive agent in the developed films.

The results demonstrate that chitosan did not decrease cell density in human keratinocytes (HaCaT), maintaining it between 102.4% and 111.9% after 24 h of treatment, even at high concentrations, compared to the quantity in the films.

## 4. Discussion

The increased degradation of films F1 and F3 at 40 °C (Figure 1, Table 1) may be related to propylene glycol in these formulations. Besides serving as a plasticizer [32,33], this pharmaceutical adjuvant has hygroscopic characteristics, acting as a humectant in the formulation [34]. Considering curcumin’s vulnerability to hydrolysis, leading to the formation of ferulic acid, feruloylmethane, and vanillin [12,35,36], propylene glycol may have favored this type of decomposition reaction. In fact, in the film where propylene glycol was absent (F2), the degradation of the active ingredient occurred only at 90 days and in a minor extension compared to the other films.

Thus, the stability studies point to film F2′s stability, even in demanding temperature conditions, and show that high temperatures become a problem for the F1 and F3 films, pointing to the need to either control the storage temperature or add stabilizers.

The release profiles of curcumin from the F2 and F3 formulations were not statistically different from each other (*p* < 0.05) after 6 h of the experiment (Figure 2). This difference increased when compared to the F1 formulation after 18 h of the experiment when the F1 formulation demonstrated entrapment of the drug in its polymeric chain. This drug retention may be related to the more compact structure of film F1. The less compact structure of films F1 and F2 exposes the hydrophilic regions, possibly leading to increased swelling. Another factor influencing film release is the swelling index, as polymer hydration leads to relaxation and interpenetration of polymer chains, increasing interaction with biological tissue [30,37].

Although the drug release between films F2 and F3 differed from F1 starting from 18 h of the experiment, all the films followed the same release kinetics model—the Weibull model. Indeed, this kinetics model commonly represents the release of substances incorporated into polymeric matrices characterized by a slow release [38,39].

Film F1 demonstrated a higher drug skin penetration despite having a lower release rate compared to the other films (F2 and F3) (Figure 3). This may have occurred due to Poloxamer^®^ 407, an amphiphilic polymer, in formulations F2 and F3. This polymer may have contributed to the formation of micelles with hydrophobic characteristics inside [40,41], trapping the hydrophobic drug and hindering the delivery of curcumin to the skin layers. The sustained release of the F1 film provides better control over drug delivery, resulting in more consistent curcumin penetration at the site of action, which seems more beneficial.

Curcumin quantification in the receptor medium of the diffusion cells remained below the quantification limit for all samples, including the free drug solution. This probably occurs because this drug penetrates the skin deeply but anchors itself through hydrogen bonding with lipid phosphate groups such as cholesterol [42].

The influence of chitosan used in the films in the drug’s penetration into the skin tissues is quite evident. It may occur because the chitosan increases cellular permeability, reversibly affecting both the paracellular and intracellular pathways of epithelial cells and promoting the depolymerization of F-actin and the dissolution of the ZO-1 protein in tight junctions, facilitating their opening and promoting higher drug delivery to these tissues [43]. These results demonstrate that the prepared films are promising delivery systems for transporting curcumin to its site of action.

The reduction in viability of the MeWo cell lineage by curcumin (Figure 4) may be attributed to significant inhibition of growth or apoptosis through cell cycle arrest in the G2/M phase and DNA fragmentation [44]. This drug also operates through the negative regulation of inducible nitric oxide synthase (iNOS) mRNA, inhibiting vasodilation present in physiological angiogenesis or the inhibition of iron-containing enzymes, potentially causing DNA fragmentation. Another mechanism of curcumin’s action to inhibit the proliferation of human melanoma cells involves increasing levels of p53, p21^Cip1^, p27^Kip1^, and CHK2 (growth inhibitors), and decreasing levels of DNA-PKcs and NFκB (cell survival factors) [44,45]. Furthermore, the IC_50_ values obtained are similar to those found in the skin permeation assay. Therefore, the amount that reaches the site of action is likely sufficient to trigger the therapeutic response, although future clinical studies should confirm such results.

The treatment of curcumin on MeWo cells was also evaluated in the presence of ionizing radiation (4, 8, or 12 Gy). The 4 Gy dose was selected because multifraction regimens are most commonly used in clinical radiotherapy. However, since melanoma is traditionally regarded as a radioresistant tumor, a higher dose of 12 Gy was also applied, along with an intermediate dose of 8 Gy [46]. Figure 5 illustrates cellular viability 24 h after treatment. No decrease in cellular viability was observed 24 h after radiation application alone (4, 8, or 12 Gy). Regarding treatment with curcumin alone, there was a decrease in cellular viability compared to treatment with radiation alone (*p* < 0.01 for 4 Gy, and *p* < 0.001 for 8 and 12 Gy). Also, there was a difference between curcumin alone compared to the control.

The absence of a decrease in cellular viability with radiation alone can be explained by the cells’ inherent high capacity for repairing sublethal DNA damage caused by irradiation [47,48]. When applied in sublethal doses, radiation may increase the risk of metastases by elevating the hypoxic fraction and hypoxia-induced positive regulation. This behavior underscores the need for dose fractionation, including more significant fractions than conventional ones [48].

Radiation can directly impact cellular components, causing double-strand DNA breaks through the induction of free radicals or reactive oxygen species in target tissues [49]. Although the results demonstrate lower cell viability from radiotherapy at doses of 8 and 12 Gy when combined with curcumin treatment, there is no synergistic effect when combined with radiation therapy compared to curcumin therapy alone. It suggests that curcumin treatment may be more effective than single doses of ionizing radiation. Therefore, combining curcumin treatment with radiation therapy for this cell lineage would have no significant benefits under the evaluated conditions.

Finally, the irritability test suggests the formulation’s safety concerning irritative potential (Figure 6), which is crucial for this type of formulation considering its prolonged contact with healthy tissues on the skin for several hours. Regarding the toxicity of chitosan alone, it may classified as a non-cytotoxic substance based on its non-observed effect on the viability of the human keratinocytes, as shown in Figure 7 [28].

## 5. Conclusions

In conclusion, the films prepared with chitosan have proven to be stable at room temperature and exhibit characteristics compatible with cutaneous applications. They demonstrate controlled drug release, exhibit no signs of irritability, are safe, and enable drug penetration into the deeper layers of the skin, ensuring the local distribution of the phytochemical. Cellular assays indicate that the estimated drug amounts penetrating the tissues would effectively reduce the cell viability of a metastatic tumor lineage (MeWo), surpassing the response achieved with single doses of radiotherapy. Therefore, bioadhesive films produced from chitosan containing curcumin appear to be a promising alternative for skin cancer topical treatment. Future in vivo clinical studies should confirm these findings.

## Figures and Tables

**Table 1:** Stability results regarding drug content, thickness, and pH for films F1, F2, and F3 at 0, 7, 30, 60, and 90 days under room temperature (RT) and 40 °C. Results are presented as a mean of a triplicate ± standard deviation.

Time	Condition	Weight (mg)			Thickness (mm)			pH		
		F1	F2	F3	F1	F2	F3	F1	F2	F3
Initial	RT	2384.0 ± 49.6	2167.5 ± 35.2	2790.2 ± 84.8	0.32 ± 0.04	0.25 ± 0.03	0.35 ± 0.03	5.5 ± 0.1	5.3 ± 0.1	5.3 ± 0.2
	40 °C	2361.3 ± 18.2	2137.2 ± 22.6	2799.0 ± 84.3	0.33 ± 0.05	0.24 ± 0.04	0.35 ± 0.04	5.5 ± 0.1	5.3 ± 0.1	5.3 ± 0.2
7 days	RT	2383.8 ± 84.8	2167.3 ± 49.9	2790.0 ± 35.3	0.31 ± 0.07	0.25 ± 0.02	0.34 ± 0.04	5.5 ± 0.1	5.4 ± 0.1	5.5 ± 0.1
	40 °C	2360.1 ± 18.2	2136.7 ± 22.6	2798.6 ± 84.3	0.33 ± 0.04	0.26 ± 0.04	0.36 ± 0.03	5.4 ± 0.1	5.3 ± 0.1	5.4 ± 0.1
30 days	RT	2383.4 ± 84.4	2167.3 ± 49.5	2789.5 ± 35.9	0.33 ± 0.03	0.24 ± 0.05	0.34 ± 0.04	5.5 ± 0.1	5.4 ± 0.1	5.4 ± 0.1
	40 °C	2357.7 ± 18.7	2135.6 ± 22.1	2796.9 ± 83.8	0.33 ± 0.02	0.27 ± 0.07	0.34 ± 0.04	5.3 ± 0.1	5.3 ± 0.1	5.3 ± 0.2
60 days	RT	2383.2 ± 84.5	2166.8 ± 49.3	2786.1 ± 36.0	0.33 ± 0.02	0.26 ± 0.02	0.36 ± 0.04	5.4 ± 0.1	5.4 ± 0.1	5.4 ± 0.1
	40 °C	2354.8 ± 20.8	2134.9 ± 22.6	2796.0 ± 83.9	0.32 ± 0.03	0.26 ± 0.06	0.35 ± 0.04	5.4 ± 0.1	5.4 ± 0.1	5.4 ± 0.1
90 days	RT	2383.0 ± 87.5	2165.5 ± 49.9	2783.7 ± 36.9	0.31 ± 0.04	0.24 ± 0.03	0.36 ± 0.04	5.4 ± 0.1	5.3 ± 0.0	5.3 ± 0.1
	40 °C	2352.8 ± 20.8	2133.8 ± 22.8	2794.9 ± 84.4	0.30 ± 0.06	0.26 ± 0.07	0.36 ± 0.05	5.5 ± 0.1	5.3 ± 0.1	5.3 ± 0.2

## Data Availability

The original contributions presented in the study are included in the article. Further inquiries can be directed to the corresponding authors.
